# Using Negotiated Joining to Construct and Fill Open-ended Roles in Elite Culinary Groups

**DOI:** 10.1177/0001839214557638

**Published:** 2015-03

**Authors:** Vaughn Tan

**Affiliations:** 1 University College London

**Keywords:** adaptation, membership processes, negotiated joining, role ambiguity, absorptive capacity, innovation, elite groups, culinary groups, uncertainty, contingency theory

## Abstract

This qualitative study examines membership processes in groups operating in an uncertain environment that prevents them from fully predefining new members’ roles. I describe how nine elite high-end, cutting-edge culinary groups in the U.S. and Europe, ranging from innovative restaurants to culinary R&D groups, use negotiated joining—a previously undocumented process—to systematically construct and fill these emergent, open-ended roles. I show that negotiated joining is a consistently patterned, iterative process that begins with a role that both aspirant and target group explicitly understand to be provisional. This provisional role is then jointly modified and constructed by the aspirant and target group through repeated iterations of proposition, validation through trial and evaluation, and selective integration of validated role components. The initially provisional role stabilizes and the aspirant achieves membership if enough role components are validated; otherwise the negotiated joining process is abandoned. Negotiated joining allows the aspirant and target group to learn if a mutually desirable role is likely and, if so, to construct such a role. In addition, the provisional roles in negotiated joining can support absorptive capacity by allowing novel role components to enter target groups through aspirants’ efforts to construct stable roles for themselves, while the internal adjustment involved in integrating newly validated role components can have the unintended side effect of supporting adaptation by providing opportunities for the groups to use these novel role components to modify their role structure and goals to suit a changing and uncertain environment. Negotiated joining thus reveals role ambiguity’s hitherto unexamined beneficial consequences and provides a foundation for a contingency theory of new-member acquisition.

Organizations cannot grow and develop without acquiring new members. Existing theory frames the systematic acquisition of new members as a selection problem in which a target group chooses from a pool of aspirants to optimize fit between the chosen aspirant and a predefined role (Jovanovic, 1979; Mortensen and Pissarides, 1999; Ployhart and Schneider, 2002). But as organizations face more uncertain external environments (Christensen, 1997; Lester and Piore, 2004; Wheatley, 2006; Stark, 2009), systematic growth poses a challenge that existing theory cannot resolve. Existing theories based on selection processes assume that organizations have or can develop predefined roles with which to acquire new members. Because organizations operating in uncertain environments often cannot fully predefine the roles their new members will have to perform (reviewed in Singh, 2008), these roles are necessarily emergent and open-ended. Consequently, existing theories cannot—and were not intended to—fully address situations in which organizations must acquire new members whose roles are open-ended or the dynamics of membership in elite groups that must be staffed from elite aspirant pools and that face uncertain future demands, for instance, partnership groups within professional service firms, tenured faculty ranks within prestigious academic departments, and top management teams within firms operating in rapidly changing industries.

These observations generate the broad question that motivates this study: What systematic processes and practices do such organizations use to acquire new members when their roles are open-ended and mostly non-predefined? I answer that question by documenting the previously unexamined process of negotiated joining in a setting in which external uncertainty often necessitates open-ended roles for new members: elite groups working in cutting-edge, high-end cuisine. I begin by examining previous research on selection and job definition to develop a theoretical framework for understanding how organizations might construct and fill open-ended roles. I then present a process model of negotiated joining and its distinctive characteristics, subprocesses and associated practices, and enabling conditions.

## Constructing and Filling Emergent, Open-ended Roles

A member’s role in an organization consists of the functions he or she performs: formal and established functions (the job specification), informal and emergent functions, and self-defined and socially defined functions (Ilgen and Hollenbeck, 1991; Ilgen, 1994). Selection theory assumes and prescribes that new members’ roles are stable (Ilgen and Hollenbeck, 1991; Ilgen, 1994) and that target groups create roles for new members before roles are matched with aspirants (Jovanovic, 1979; Stewart and Carson, 1997; Mortensen and Pissarides, 1999; Singh, 2008). Aspirants are assumed to select and apply for the roles they want to perform in specific target groups (Granovetter, 1995; Judge and Cable, 1997), and these target groups are assumed to acquire new members by selecting for best fit from among the aspirants that they have attracted (Jovanovic, 1979). This study concentrates solely on the latter: the processes target groups use to systematically acquire new members.

In selection, the target group’s goal is to optimize two types of new-member fit: cultural fit (O’Reilly, Chatman, and Caldwell, 1991; Van Vianen, 2000) and simple fit with a predefined role (Arthur et al., 2006). The greater the fit between the selected individual and the predefined role, the more successful the target group is at selection. Selection is conceptualized as a linear process of applying appropriate selection methods (summarized in Stewart and Carson, 1997; Schmidt and Hunter, 1998) to come to a selection decision. These selection methods cannot work without a predefined set of criteria for deciding between aspirants to a given predefined role. Due to this fundamental requirement and assumption, research and theory in selection were not intended to—and cannot—address situations in which roles are open-ended. Theories of how jobs are defined and shaped, however, provide some limited insight into how emergent, open-ended roles might be constructed and filled. Three perspectives in this area are particularly relevant: research on idiosyncratic jobs, on job assembly, and on job crafting.

### Idiosyncratic jobs

Selection has traditionally been presented as a systematic, managerially driven, top-down process. Research on idiosyncratic jobs offers an alternative view that encompasses two phenomena: (1) opportunistic hiring into jobs specifically created to fit new members and (2) evolution of incumbents’ jobs during their tenure (Miner and Estler, 1985; Miner, 1987). These jobs are identified as idiosyncratic because they are highly personalized and also because they result from individual action by hiring managers (in the case of opportunistic hiring) and incumbents (in the case of role evolution). Idiosyncratic jobs have been found to be associated with increased mission ambiguity and resource uncertainty and are thought to be the product of adaptive actions by managers and incumbents, possibly representing “intelligent action in the face of uncertainty and ambiguity” (Miner, 1987: 334). This perspective suggests that open-ended roles can be constructed not only from the top down by managers acting on behalf of the firm but also from the bottom up by individuals acting out of self-interest (Miner, 1990).

### Job assembly

Whereas selection requires predefined integral roles, job-assembly theory suggests that jobs are put together out of multiple tasks (Cohen, 2012). These tasks can enter groups through active search and passive reception; they can also be generated by the learning that occurs in the course of work or be part of existing ideas about jobs. Job-assembly theory thus indicates that job design is an “evolving response to the ebb and flow of information and interactions” within organizations (Cohen, 2012: 450). This perspective suggests that the construction of open-ended roles is likely to be an iterative and evolutionary process operating on the components of roles rather than on monolithic roles.

### Job crafting

In contrast to the static roles that selection depends on, job-crafting theory proposes that roles are plastic and can be shaped by role incumbents. Research in this area documents how employees use interactions with coworkers to modify the task, cognitive, and relational boundaries of their prescribed jobs “to create work with which they are more satisfied” (Wrzesniewski and Dutton, 2001: 181). This perspective suggests that role construction can be driven by individuals motivated to expend effort to modify their roles to increase their satisfaction. It also indicates that a significant part of role construction happens through interactions and interpersonal sensemaking and negotiation (Wrzesniewski, Dutton, and Debebe, 2003).

These three theoretical perspectives suggest that open-ended roles are likely to be constructed and filled through iterative and evolutionary processes that (1) operate on role components rather than on monolithic roles, (2) are motivated by individuals’ desire for satisfying work, (3) are responsive to external uncertainty and ambiguity, and (4) happen in the course of interpersonal interaction. The interactive, constructive nature of these processes is consistent with theories of organization around emergent relationships (Stewart and Carson, 1997), of the continuous social interpretation of organizational components such as roles (Weick, 1995; Weick, Sutcliffe, and Obstfeld, 2005), and of the potential for negotiation to generate novel social understandings interactively (Mead, 1934; Strauss, 1978; Fine, 1984). Yet the extant research on job crafting, job assembly, and idiosyncratic jobs remains insufficient to explain how an organization that must construct and fill new open-ended roles can do so systematically.

Existing research depicts emergent jobs as the product of individual, uncoordinated action without top-down organizational involvement (Miner, 1987, 1990), though the widespread nature of emergent roles in some settings suggests that they can also result from systematic processes stemming from intentional organizational action. Job assembly focuses on formal tasks, though non-task role components (Parsons, 1956) are likely to be especially important components of an individual’s role in a group facing high levels of external uncertainty (Ilgen and Hollenbeck, 1991). Job crafting focuses on employees’ motivations and actions and tells us little about how job crafting affects (and is affected by) other members and the group as a whole, though these contextual effects are likely to be significant (Berg, Wrzesniewski, and Dutton, 2010). Finally, all three perspectives consider only role-shaping actions taken by employees who are already members of the organization, leaving aspirants out of the picture.

This paper attempts to answer some questions left unasked by previous research: What systematic processes do organizations use to acquire new members when task and non-task components of their roles are open-ended, not predefined? What processes allow these open-ended roles to be constructed and filled, and how do they affect the roles of both aspirants and incumbents? And, finally, what practices are associated with these processes? Such questions deal with poorly characterized processes and are best addressed in settings in which the phenomena under investigation are likely to be clearly observable (Pettigrew, 1990). This study therefore begins to answer these questions in an unconventional setting (Bamberger and Pratt, 2010) in which open-ended roles are the rule rather than the exception: elite groups working in high-end, cutting-edge cuisine. Though these elite groups range from small restaurant operating teams to culinary R&D labs, open-ended roles are common to them all.

### High-end, Cutting-edge Culinary Work

Among high-end culinary groups, innovation has become a key differentiating factor and source of competitive advantage, the latest stage in the constantly evolving identity work that has characterized the institutionalization of high-end culinary work in recent history (Rao, Monin, and Durand, 2003). Because novel output is now so important, the elite groups working in high-end, cutting-edge cuisine invest considerable time and effort in innovation, often organizing their operations and service models around research and development. These elite groups work on creating new ingredients, cooking processes, and equipment, with the intent of eventually creating new dishes and dining experiences (Abend, 2011; Gopnik, 2011; Kramer, 2011).

Because their identity depends so much on being perceived as innovative, these elite groups generate novel ideas rapidly. They must also constantly respond to the work of competing groups to differentiate themselves and reach and remain at the high end of the industry. As a senior chef at a European restaurant said, “We’re not in a world where you can learn the techniques and then coast for years anymore. Now there’s, like, something new to have to think about every day. We’re running just to stay in place” (I-46).^1^ The state of the art in this industry changes so quickly that demands placed on these groups are unpredictable, making individual members’ roles difficult or impossible to define in advance—an ideal setting in which to examine how groups acquire new members with open-ended roles.

## Methods

This study focuses on how elite culinary groups—the target groups to which potential members aspire—manage new-member acquisition from their respective aspirant pools instead of how and why aspirants choose the particular target groups they apply to. Consequently, my unit of analysis is the joining process through which a stable role was constructed for and by the aspirant or, conversely, through which the target group and aspirant realized that a stable role was unlikely. I treat each individual moving through this process at a particular group as a case, analyzing the narratives within each case (Dyer and Wilkins, 1991) to describe similarities in the processes aspirants go through.

I developed the cases from in-depth, unstructured interviews with 88 individuals working in high-end cuisine and more than a thousand hours of ethnographic observation time spent with nine internationally renowned task-oriented groups in North America and Europe working in high-end cuisine. In-depth interviews and extended ethnographic observation (Becker et al., 1976; Dyer and Wilkins, 1991; Michel, 2011) and case study methodology (Yin, 2002; George and Bennett, 2005) are appropriate for investigating and theorizing about processes such as negotiated joining that are not yet thoroughly researched (Edmondson and McManus, 2007). These cases incorporated perspectives from both the focal aspirant and multiple incumbents from the target group, and the set of cases contained both cases that resulted in stable roles for the aspirants and those that did not. This multi-perspective, multiple-case analysis was an attempt to mitigate the tendency to misjudge the extent to which a single case is generally representative (Tversky and Kahneman, 1986).

### Data Collection

I acquired respondents and observation sites using snowball referrals (as described in Biernacki and Waldorf, 1981) from initial contacts who were prominent in and familiar with the industry. I interviewed 88 respondents in total, in many cases in multiple interviews. A few were dedicated interviews, but most were real-time interviews conducted ad hoc in the course of work or casual conversation (Barley and Kunda, 2001).

Interview times with respondents ranged from 45 minutes to over three hours, and 33 respondents were prospective members (“aspirants,” labeled A-1 through A-33 in the following analysis), while 55 respondents were current members of a group (“incumbents,” labeled I-1 through I-55). Of the 33 aspirants, 14 were stagiaires—people working temporary and usually unpaid stints at observation sites. Respondents’ locations were about evenly divided between North America (48) and Europe (40), and their gender and age makeup was approximately representative of cutting-edge, high-end culinary groups: 74 respondents were male, and 68 were in their 20s and 30s (two were in their late teens, and 18 were in their 40s and 50s).

I initially recorded interviews whenever my respondents permitted me to do so, taking notes during the interview and transcribing selectively afterwards, but I soon discovered that kitchen background noise made audio recordings of interviews largely unusable. I eventually abandoned the recording apparatus whenever I was interviewing someone in a working environment and relied instead on pen-and-paper field notes or, at some sites, field notes written directly into an on-site lab computer. I combined partial interview transcripts (when available) with notes written up during and after interviews and analyzed them for instances of joining. At several points in the analysis, I contacted respondents to ask clarifying questions about the setting.

Over a four-year period, I gained research access to nine groups and visited each group several times, each visit lasting between ten days and six weeks. Table 1 summarizes information about these field sites, including observation time spent at each location. Of the nine groups, five operated a single restaurant, two conducted R&D for restaurant groups, and two conducted R&D independently—reflecting three distinct models of high-end, cutting-edge culinary work.

**Table 1.** Observation Sites

Site name	Description	Observation time (approx.)
Bharat Peruano	15-person team running a restaurant featuring subregional cuisines from India and Peru (U.S.).	130 hrs.
Montano	6-person team running a critically acclaimed high-end restaurant featuring innovative multicourse tasting menus (U.S.).	130 hrs.
Rubicon	12-person team of chefs supporting development of menu items across 12 restaurants and new high-end restaurant concepts around the world (U.S.).	160 hrs.
Pacifica	6-person team running an established, critically acclaimed, small restaurant featuring innovative, technical multicourse tasting menus (U.S.).	90 hrs.
Walsall	6-person team supporting a Michelin-starred restaurant and six other restaurants, and content development for licensing deals, television, and books (Europe).	60 hrs.
Kerberos	22-person team running an R&D lab primarily developing content for books and new media (U.S.).	75 hrs.
Erebus	3-person team running a culinary R&D lab with sporadic production and research partnerships with industry and restaurants (Europe).	100 hrs.
Øresund	65-person team running a critically acclaimed, Michelin-starred restaurant featuring innovative, technical multicourse tasting menus (Europe).	350 hrs.
Turnstone	28-person team running a critically acclaimed, Michelin-starred restaurant featuring innovative, technical multicourse tasting menus (Europe).	50 hrs.

I arranged my visits to coincide as much as possible with the presence of aspirants at various points in the joining process. I spent more than a thousand hours on site (approximately 14 working weeks of 14- to 18-hour days) observing these groups and their incumbents in the course of their work, in the process of evaluating aspirants, and in their pre- and post-work social interactions. Nearly every respondent had experience as both an aspirant and an incumbent. Of the 33 respondents who were aspirants during my data collection, 32 were working at field sites; I was able to observe them in the negotiated joining process. Nineteen of them achieved a stable role at their respective sites, while 13 abandoned the process. As often as was possible, I spoke with both aspirants and incumbents to obtain both perspectives about aspirants’ movements through the negotiated joining process. While on site, I took digital photographs and kept short-form notes in a notebook and on-site computer. I wrote nightly long-form syntheses of field notes, weekly summaries, and analytic memoranda at the end of each field visit (approximately as described in Emerson, Fretz, and Shaw, 1995).

### Data Analysis and Reduction

Data coding and analysis occurred in tandem, in two major stages. The first stage was connected to data collection. My fieldwork began as a series of in-depth interviews from which I developed some retrospective cases—incumbents’ accounts of their previous experiences as aspirants. Preliminary analysis of these cases revealed the unusually protracted nature of recruitment in these groups. I then returned to the field over the course of four years to observe recruitment processes in real time, focusing on data relating to the schemas and frames emerging from the analysis of interview data. These first-stage codes clustered around key themes emphasizing the importance of provisional roles, the negotiated nature of stable roles, and the extended process of developing a role in a target group. I used this rough coding in the field as I collected the observational data and conducted new interviews. While at these sites, I continued to interview new respondents and re-interview previous respondents. The cases I developed out of field observations and new interviews validated and added nuance to the retrospective cases.

I began the second stage of coding and analysis after completing fieldwork. I reviewed the cases to identify similarities in process and practice across them. From this, I produced a list of codes for the sequential subprocesses (proposition, trial, evaluation, and integration) and associated practices described below. My subsequent analysis of the patterning of these codes across all cases revealed the sequential ordering of the subprocesses and the iterative nature of negotiated joining as a whole process. At several points in both stages of the analytic process, I asked people working in similar settings who were not part of my respondent set to review and validate my analyses and interpretations of interview and observational data.

## Negotiated Joining

My findings document how negotiated joining allowed aspirants and target groups to surmount two challenges that resulted from open-ended roles that were mostly non-predefined. The first challenge was learning if a mutually desirable role—a set of role components that the aspirant would find desirable to undertake and that would satisfy the target group’s emerging set of needs—was likely for a given aspirant–target group combination. The second challenge was constructing such a mutually desirable, stable role. Negotiated joining had distinctive characteristics, subprocesses and practices, and enabling conditions.

### Distinctive Characteristics

Aspirants had to apply to the target groups and be selected to participate in negotiated joining. But respondents said that this initial selection process by the target group involved a small set of selection criteria and that selection did not mean that the aspirant had been hired into a permanent job. In fact, the opposite was true: incumbents at every target group said that they screened résumés of potential aspirants with “some idea of what we’re looking for” but needed to spend time working with the aspirant “to give us an opportunity to find out more about him but also so that we give him time to learn about us and tell us how he thinks he’ll fit in with what we do” (I-34). They described selection in this setting as a precursor to “the real work of joining the group and making a place for yourself” (I-10).

Target groups used this first selection process to choose aspirants to enter a consistently patterned, systematic negotiated joining process with five distinctive characteristics: (1) aspirants’ initial roles were mutually explicitly acknowledged to be provisional, (2) aspirants and target groups both emphasized the importance of learning whether a mutually desirable eventual role was likely, (3) aspirants and target groups both emphasized the importance of constructed role fit, (4) mutually desirable roles were constructed through iterative rounds of role negotiation, and (5) both aspirants and target groups framed abandoned negotiated joining processes as valuable learning experiences instead of failures. Table 2 presents evidence for these key characteristics of negotiated joining.

**Table 2.** Evidence for Distinctive Characteristics of Negotiated Joining*

Distinctive characteristic	Evidence
Initially provisional roles	“Of course [the new member’s role] is a bit floating, a bit [*makes hand-waving gesture*]—I know a little bit what I want the new person to do but stuff happens so fast around here that it’d be silly to try to put too much structure in at the start. It’s better to wait and see.” (I-17)
Emphasis on learning whether a mutually desirable eventual role was likely	“You have to figure out a job that you’re happy with that [the target group] is also happy with. That’s the important thing about it. If you can’t make it work out, it’s probably best to just move on.” (A-9)
Emphasis on constructed role fit	“. . . it’s not like my previous job [in software product management] where once I got the job I had pre-set expectations of what I needed to deliver and my bonus was pegged to that. . . . here, to get the job I have to build it. [The target group] doesn’t have a clear idea of what I can do for them, so I have to put that picture together and really sell it.” (A-17)
Role construction occurred through iterative role negotiation	“You don’t figure it out straight away . . . it takes a while to try things out not only for you but also for [the group] to see if you’re good at it. And of course if it’s useful for them.” (A-29)
Abandonment of negotiated joining framed as learning about preferences	“[A particular aspirant] left because we couldn’t figure out something for him to do that he found worthwhile. . . . it was clear when we were working together that he was good at the [R&D] part of things but it turned out that we couldn’t give him enough [of that work] and he didn’t really enjoy [working] service enough to make it worthwhile.” (I-12)

* I = incumbent, A = aspirant; the number following denotes an individual respondent in this study. Thus, I-17 is the 17th of 55 incumbents.

#### Roles explicitly and mutually acknowledged to be initially provisional

Target groups recognized that they did not or could not know in advance the complete set of functions they needed new members to perform. Consequently, the aspirant’s initial role was meant to be a starting point rather than the intended end state. Though the aspirant’s role was initially provisional, it was neither completely undefined nor unintentionally undefined. Many respondents described how a part of their eventual role was clearly defined from the beginning. But even when there were specific expectations about an aspirant’s eventual role, part of that role was explicitly and intentionally left open to change or definition. Respondents said that both the aspirant and the target group had to explicitly and intentionally acknowledge the initial role’s provisionality for negotiated joining to take place. When this was not the case, negotiated joining could not proceed: “. . . there wasn’t any flex for me to do anything interesting . . . they knew what they wanted from me but I wasn’t into it enough [for me] to want to stay” (A-1, recalling previous aspirant experience).

#### Emphasis on learning about the likelihood of a mutually stable role

Because roles were open-ended, neither aspirants nor target groups knew in advance exactly what a new member’s role would be or if it would be mutually desirable. Respondents said that an important aspect of negotiated joining was therefore to learn whether a mutually desirable role was likely: “. . . there’s no way to know for sure [if or what your stable role will be]; you have to be pro-active and put some time into figuring out if [a particular target group] is the place for you” (I-1).

A mutually desirable role was one the aspirant wanted to perform that was also relevant and useful to the group. Aspirants highlighted the importance of “figuring out a job that is satisfying” (A-6), while incumbents highlighted the importance of “making sure that [the aspirant] brings something useful to the table” (I-30). Crucially, a role had to fulfill both criteria simultaneously to be mutually desirable and thus stable: many respondents echoed one incumbent’s explanation that “what’s important . . . is getting to the point where [both the group and the aspirant] know that we’ve got a good thing going” (I-22).

#### Emphasis on constructed role fit in addition to simple role fit and cultural fit

Respondents said that negotiated joining helped them identify both cultural fit and simple fit with predefined roles: “. . . spending time working together is essential to see if we can work together, if [the aspirant] works in the same way that we do” (I-11). But they emphasized that constructed role fit was the most important part of negotiated joining. Respondents described their roles as modular assemblages of role components and “figuring out a role” as involving putting role components together to produce a mutually desirable role (I-2). These roles were thus tailored to both the aspirant and the target group. These role components were sometimes novel to the group but could also be modifications of existing role components that were then assembled into novel configurations. Negotiated joining allowed aspirants and target groups to propose and validate both novel role components and novel configurations of existing role components. Respondents said that most of the stable roles constructed were novel in that each stable role was the first instance of that particular configuration of role components in the target group. For example, one incumbent, describing a colleague’s role, said,

[He’s] the first sous chef we’ve ever had who spends so much time managing our [hundreds of] supplier relationships . . . he spends very little time in the [service] kitchen compared to the other sous [chefs]. At first [the head chef] didn’t think it would be worth it to spend a person on this job but he just started doing the work anyway and it quickly became obvious that having one person manage all of [the suppliers] makes a huge difference. (I-36)

This role combined new and existing role components in a configuration novel to the group, and the role was stable because it was both personally satisfying to the aspirant who eventually filled it and relevant to the group’s goals.

#### Role construction through iterative rounds of mutual role negotiation

Respondents said that aspirants’ roles gradually accumulated out of only validated role components over the course of multiple rounds of negotiation. Role negotiation was consistently described as a mutual process that operated through routine work interactions, with the aspirant acting to “show [the target group] that you’re good enough to perform [a particular role component]” (I-52) and the target group acting to evaluate the aspirant’s performance of that role component and “see if [the role component] is useful [to the target group]” (I-2). When aspirants I observed managed to construct a stable role (19 cases out of 32), the role only stabilized after repeated sequences of negotiation, most of which resulted in a validated role component that was then incorporated into the aspirant’s stable role.

#### Abandonment of negotiated joining framed as a learning experience

In 13 of the 32 cases, the negotiated joining process was abandoned after a sequence of mostly unsuccessful attempts to negotiate role components—in 12 of these 13 cases, the aspirant made the decision to abandon the process. Eighty-three out of 88 respondents said that they had gone through negotiated joining at least once in their careers, and those who had been in the industry longer said that they had abandoned multiple negotiated joining processes. Across all respondents, however, these abandoned negotiated joining processes were not framed as failures. Instead, respondents reported that abandoned negotiated joining processes allowed them to learn about their skills and inclinations as well as the types of groups they would likely be happy in. A senior incumbent with extensive industry experience said that “you can only learn about the work you want to do by trying it out. Most of the time, it’s not quite right . . . you have to be disciplined about learning what you can from the situation and moving on” (I-16). This was also true from the target group’s perspective: “. . . even after years of doing this we still don’t know what kind of person will work well here. . . . So you have to keep an open mind as a [group]. But the more people come through, the more we know about what kind of person probably won’t work so well here” (I-52).

Both aspirants and incumbents said that they interpreted abandonment of negotiated joining as indicating either (1) poor fit with the target group’s existing culture or with predefined role expectations or (2) that the aspirant and/or the target group believed that no mutually desirable role could be constructed:

I went into [a previous target group] knowing that they were trying to build an R&D team but weren’t sure exactly what kind of work they were trying to do. While I was there, we tried [a few different approaches] but in the end the only things [that turned out to be viable for them] were things that I found fundamentally uninteresting. (A-18, describing an earlier abandoned negotiated joining process)

Respondents said that selection was relatively unimportant in this context compared with negotiated joining. Moreover, selection could not be substituted for negotiated joining because it was impossible to fully or mostly predefine a new member’s role:

. . . when they hired [a new R&D head from a traditional R&D management background], he asked us all to write out detailed job descriptions to streamline operations and become more efficient. That’s when everything really began to go to shit. It’s very satisfying at first to have these long lists of roles and responsibilities. It definitely feels very efficient and like you’re getting something done. But does it work in real life? No. There’s no point being efficient and hiring for a very detailed job description if the job changes every day. That’s being efficient about the wrong things. (I-37)

Respondents consistently reported that negotiated joining was an iterative process—distinct from selection—that mediated the transformation of an initially provisional role into a stable, mutually desirable one, or the discovery that no stable role was likely. My analysis revealed the subprocesses, practices, and enabling conditions of negotiated joining.

For aspirants, negotiated joining was experienced at any given moment as potential involvement in and resolution of multiple role-component negotiations. This role negotiation consisted of and was interwoven with the daily routines of work. Through work interactions like preparing a consommé, butchering a fish, jumping in during a busy service, volunteering to do inventory, and countless others, aspirants and incumbents engaged in a constant stream of negotiations about various role components—some concrete, some abstract, some major, some minor.

Negotiated joining entailed four conceptually distinct subprocesses that were consistently patterned and sequentially ordered and were associated with specific practices: (1) the proposition of role components, validation of proposed role components through (2) practical trial, followed by (3) evaluation of trial results, and (4) selective integration of only validated role components into the aspirant’s role. The process moved through the subprocesses in sequence, each iteration potentially adding validated role components to the aspirant’s role. The iterations continued until a mutually desirable role was constructed or the aspirant decided to abandon the process and leave the group. Table 3 presents evidence for the four subprocesses and their associated practices.

**Table 3.** Evidence for Practices Associated with Negotiated Joining Subprocesses

Practice	Evidence

**Proposition**	

Problematizing routine work	“. . . the best thing to do is to keep thinking about problems. . . . It’s about training yourself to have that mindset of looking at everything [the target group is doing] and seeing past the routine. . . . that’s your chance to show us that you can make our kitchen work better.” (I-17)
Integrating aspirants into incumbent workflows	“. . . it’s hard not to notice if someone is really good or bad at doing something [if you’re working in the same room]. Like, [a particular stagiaire] is a complete mess. . . . all the wrong instincts for service. . . . But he’s actually really useful if there’s a question like, I don’t know, how do I make this foam more stable? We were working on something like that the other day and he’s got an instinct for that stuff.” (I-30)

**Trial**	

Clearly stated trial parameters	“. . . [trials] work best when it’s clear what you’re testing. [As an aspirant] I figured out quickly to set up [the trial] so that it’s obvious what I’m doing and how it’s affecting the result . . . if I’m going to be evaluated for something, it better be the right thing.” (I-25)
Scheduling frequent and routine trial opportunities	“I feel a lot more comfortable bringing stuff for feedback [when everyone is planning to be there anyway]. It helps that [the feedback session] doesn’t feel like a super special thing where the stakes are high.” (A-24)

**Evaluation**	

Using mutually understood evaluation standards	A-30 has been asked to work on developing a sauce for a new dish. I-37 tells A-30 to focus on the texture of a sauce he is developing: “Remember that egg custard [the pastry chef made] last week? Aim for that kind of texture.” As A-30 worked, he could rapidly eliminate many development pathways from consideration because he had an accurate and precise idea—from a shared experience of the desired evaluation standard—of the desired outcome. (Field notes from observation at a European restaurant)
Large group evaluations	The role component A-33 was trialing during service was his ability to execute many orders accurately under time and psychological pressure. At the post-service debriefing, the head chef’s evaluation of A-33’s performance was that it was “a disaster,” due to his lack of “awareness of [the progression of] service.” During the large group debriefing, other incumbents provided information the head chef did not have about mitigating circumstances beyond A-33’s control—this changed the group’s prevailing interpretation of his performance to a potentially favorable one. (Field notes from observation at a European restaurant)
Involving focal and non-focal participants in evaluations	“. . . having other [non-focal] people [in the group] be part of the feedback process is very useful . . . [because they can] see what kinds of questions we’re asking and what the mindset and our priorities are when we’re trying to refine a recipe. That’s hard to learn except by failing and watching other people fail.” (I-17)

**Integration**	

Gradual commitment	“It’s not a ‘you’re in, you’re out’ situation [with this role component]. It’s got to be baby steps. This last batch of recipes tested out but if [the head chef] asked me to go out now and do these on my own I wouldn’t be confident.” (A-29)
Having as many incumbents as possible attend evaluations	“I always make people come to group feedback sessions if they are in [the restaurant]. . . . You can’t learn if you’re not present and if you don’t learn, you can’t change how you do your job.” (I-49)
Having non-focal incumbents participate in evaluations	“. . . when you have to give feedback on someone, you can’t just say ‘I like it’ or ‘That sucks.’ Gotta give reasons, you know, justifications . . . having to explain why something [the aspirant] did is good or bad, it helps convince other people in the group . . . and it makes you think harder about the feedback too.” (I-6)

### Subprocess: Proposition

#### Problematizing routine work

In the initial stage of each iteration of negotiated joining, either the aspirant or the target group developed and then proposed a role component that might—conditional on successful testing, evaluation, and integration—become part of the aspirant’s eventual role. Aspirants were motivated to develop and propose relevant role components out of a desire to convert an initially provisional role into a stable one, thus obtaining membership in a personally aspirational group. Respondents said that a role component that was relevant to the target group was more likely to be validated and contribute to the aspirant obtaining membership: “. . . it would be great to [work] here but it can’t just be like a lame job, yeah? It has to be something that keeps me excited but it has to work for them too” (A-5). Respondents reported that aspirants who were in the habit of problematizing the target group’s system of routines and norms were more likely to see functional gaps in their target groups and were thus able to identify more relevant role components to propose. Respondents also said that role components could emerge serendipitously: “. . . [my present role] is just how we split up the work, and then we realized it was working quite well and . . . we kept on splitting up the work the same way” (I-34). Interactions during routine work allowed aspirants and incumbents to identify and propose role components like these.

#### Integrating aspirants into incumbent workflows

At the three sites where incumbents and aspirants worked in close proximity (though rarely interdependently), incumbents seemed to propose role components for aspirants more frequently than at the three sites where incumbents’ and aspirants’ workflows were not as well-integrated. The more interaction, the more opportunities incumbents had to observe aspirants working, identify potential role components, and propose them for specific aspirants: “. . . if I see that [a particular aspirant] is rocking out [a particular set of tasks], I’m going to keep sending them his way” (I-49). Constant interaction also encouraged aspirants to present their abilities honestly: respondents said that being closely observed was “not a situation where you can try to pretend to be good at doing something because you’re going to get called on it” (A-29). Aspirants in highly integrated workflows were thus more likely to act in ways that would allow incumbents to make well-informed role propositions for them. The practice of integrating aspirants into incumbents’ workflows thus appeared to support effective incumbent-driven role proposition for aspirants.

Many role components proposed for or by aspirants were already performed by incumbents, but aspirants sometimes proposed role components that were entirely novel to the target group. For instance, I observed an aspirant (A-3) at a U.S. restaurant use his previous work experience to propose a role component as the group’s first bread-and-yeast specialist. These novel role components sometimes had broader implications for other incumbents and the group as a whole—as I discuss below in describing the integration subprocess—but the end result of the proposition subprocess was that a role component was proposed either for or by the aspirant. This initiated the next subprocess in negotiated joining: practical testing of the proposed role component.

### Subprocess: Trial

Proposed role components were validated in action, through trial and evaluation of the aspirant’s performance of the role component in the context of group work and group goals. Trials of role components took many forms but nearly always looked “just like regular work” (I-14). Any work interaction could function as a trial if it simultaneously provided an opportunity to evaluate an aspirant’s performance of a role component and the aspirant and target group incumbents treated it as a trial to be evaluated. Trials were either implicit or explicit: a junior and a senior chef working on a fish soup recipe deciding to compare their preferred methods of clarifying fish stock to see which one worked better (an implicit trial observed at a European restaurant), or a junior chef demonstrating to more senior colleagues what he asserted was a more efficient method of filling a large number of micro-pipettes with aromatized oil (an explicit trial observed at a U.S. restaurant group). In both cases, the interaction served both the task at hand (clarifying stock or filling pipettes) and evaluation of a proposed role component.

Practical trials of role components allowed the aspirant and the target group to learn about the performance of a proposed role component through concrete experience instead of mere assertion. Consequently, target groups could and did use them to evaluate an aspirant’s fit with a predefined role. But trials performed more than just this selection-oriented function: they were also used by the target group as a source of information about those aspirants’ suitability for novel role components or novel configurations of role components.

#### Clearly stated trial parameters

Trials were rife with misunderstandings. Aspirants and target group incumbents didn’t always share the same assumptions about when a work interaction was a trial and when it was not. Respondents often mentioned aspirants who “weren’t treating [the work] as a chance to prove that they’re capable” (I-5) and viewed these as “wasted opportunities” on the part of the aspirants concerned (A-4). Also, aspirants and incumbents did not always have the same understanding about the nature of the role component being tested: “I always try to be clear about what the point [of the trial] is but sometimes it just doesn’t get across [to the aspirant]” (I-9, commenting on a just-completed trial). Aspirants and incumbents both said that making the effort to clearly state trial parameters reduced the occurrence of both types of misunderstandings.

#### Scheduling frequent and routine trial opportunities

Frequent, routinely scheduled trial opportunities seemed to reduce aspirants’ perceived and actual barriers to committing to a trial of a role component—this practice seemed to increase the total number of trials each aspirant undertook and accelerate the trial subprocess. Five of the nine sites had weekly recurring meetings during which all aspirants and incumbents would meet regardless of whether any trials had been prearranged, and any aspirant or incumbent could bring work (such as a prototype of a recipe under development) to these meetings for feedback from other attendees. An aspirant in one of these groups said that “the bar for showing something [for feedback] seems low. Everyone’s going to come [to the session] anyway and lots of other people are showing stuff too . . . there’s not much pressure” (A-1). In contrast, aspirants at the other four sites had to organize trials ad hoc. Respondents at these latter sites said that the logistical challenges of scheduling a trial and “getting [all the incumbents together] to give feedback [on a trial] is a massive pain in the ass” (A-30). They were consequently more reluctant to propose and test role components and seemed to do so less frequently.

### Subprocess: Evaluation

Evaluation was conceptually distinct from trial even though the two subprocesses could (and often did) occur at the same time: evaluation entailed target group incumbents interpreting a trial’s outcome and assigning meaning and value to it. This interpretive process required incumbents to come to an agreement about the aspirant’s competence at the role component.

#### Using mutually understood evaluation standards

Evaluation was straightforward when aspirants and incumbents both shared the same understanding of the standards by which the trial performance would be evaluated, for instance, when an aspirant and an incumbent agreed to use a liqueur they had both sampled together previously as the standard for the acid–sugar balance of a new sauce on which the aspirant would be working (observed at a U.S. restaurant). Using mutually understood standards for evaluating trial performance seemed to significantly enhance an aspirant’s ability to iterate rapidly toward a desired output for any given trial and thus to improve a trial’s accuracy in testing a particular role component. Nonetheless, this practice was the exception rather than the rule—I observed it frequently at only one of the nine field sites.

Role components with more abstract standards for performance required more interpretive work. An aspirant said that “it’s not like . . . seeing if someone can break down [into usable pieces] a side of beef. I couldn’t tell you what it means to have an R&D mindset like what [the senior incumbent in the group] says we all need to have. I don’t think he could tell you either. It all depends on what the specific problem is that we’re trying to solve. So it’s also hard to show [the incumbents] that you have what it takes to do the job” (A-9). The related practices of holding large group evaluations and debriefings and of involving both focal and non-focal participants in trial evaluations were especially useful for trials of abstract role components.

#### Large group evaluations

Evaluating more abstract role components required incumbents to accumulate multiple pieces of confirming or disconfirming evidence of competence: “you can’t tell about these things from just a few [trials]. It takes time . . . you have to see if they always perform the same way in similar situations” (incumbent, chef at a U.S. restaurant). Incumbents also had to deliberate interactively and collectively assign meaning and value to trial outcomes for more abstract role components. For instance, all the senior incumbents with R&D responsibilities at a particular target group debated at length a particular aspirant’s ability to “develop new recipes in the spirit [of the group]”; these debates took place through a series of discussions, each associated with one of a series of dishes the aspirant developed and presented for tasting and feedback over the course of a few weeks (observed in a European restaurant). Collective deliberation allowed incumbents to obtain and synthesize disparate perspectives and pieces of information on the aspirant’s performance.

#### Involving focal and non-focal participants

Evaluations usually took the form of discussions of events that had taken place during service or feedback sessions on particular dishes, and they involved up to four possible types of participants: focal incumbents (who were primarily responsible for giving evaluations), focal aspirants (whose work was being evaluated), and non-focal incumbents and aspirants (who were peripheral to the particular trial being evaluated). Evaluation sessions consisted, at a minimum, of focal aspirants and focal incumbents. But sessions that also involved non-focal incumbents (three of the four types of participants) allowed information from more incumbents’ perspectives to be incorporated into the evaluation. This seemed to permit more complete evaluations of the focal aspirant’s performance. Group evaluations that also included non-focal aspirants (i.e., all four possible types of participants) were even more useful: respondents said that participating in these sessions allowed non-focal aspirants to better understand the target group’s work practices and approach, which improved their ability to propose mutually desirable role components for themselves. These latter two practices also supported the integration of newly validated role components.

### Subprocess: Integration

Integration of a validated role component occurred at three levels: that of the aspirant, the incumbent, and the group as a whole. Integration of an aspirant’s role component sometimes resulted in unintended but profound changes to the target group’s goals and role structure.

#### Aspirant-level integration: Working out parameters of a role component

Respondents said that even after role components were validated by evaluation, it “could take a while” (I-10) for aspirants to learn the parameters of a particular role component—the range of situations in which an aspirant was expected to perform that role component. This was particularly true for more abstract role components.

##### Gradual commitment

Aspirants integrated these abstract role components voluntarily, initiating additional trials of the role component in situations of increasingly significant consequence; in other words, additional iterations of negotiated joining for the same role component. Aspirants described these additional trials in similar ways: “baby steps” (A-29), “keeping the training wheels on for now” (A-14), and “easing in” (A-3). One aspirant said,

I have to come up with dishes that channel [this restaurant’s] ethos but are creative and new at the same time. It would be dumb to imagine that having [the head chef] be excited about the three or four dishes I’ve come up with [in the last few weeks] means I’m now ready to fly solo. . . . I’m going to keep doing more small projects. I have to slowly get a feel for [this group] . . . and they have to get used to my particular sensibility in food. (A-29)

This gradual commitment gave the aspirant and target group more opportunities to test a role component under different circumstances—this generated more reliable information for the target group about the aspirant’s competence and more information for the aspirant about the circumstances under which that role component might be applicable.

#### Incumbent-level integration: Modifications to incumbents’ roles

Incumbents’ roles almost always changed when a role component was allocated to an aspirant. Respondents consistently said, and I observed, that they took it for granted that incumbents’ roles were only relatively stable and might change at short notice due to an aspirant’s role negotiations. They took considerable pride in how their roles were constantly in flux and how, as one incumbent put it, “you have to stay on your toes in this business” (I-33).

When an aspirant was allocated a role component already performed by incumbents, those incumbents either relinquished that role component or shared responsibility for it with the aspirant. The evidence of competence produced by the proposition–trial–evaluation sequence was crucial to this integration and adjustment. Incumbents said that such responsibility transfers were usually non-contentious because the aspirant’s competence was demonstrated through practical trial: “the nice thing is that we can all see if [the aspirant] is better than we are at something or not. [Having gone through a series of trials and evaluations, we know] she’s got the chops to take on [a role component I currently claim]. I respect that” (I-6).

##### Having as many incumbents as possible—including non-focal incumbents—attend evaluations

Respondents said that having as many incumbents as possible attend trial evaluations promoted integration by quickly spreading personally verified knowledge about aspirants’ competence at specific role components among incumbents. Respondents also said that the practice discussed earlier of non-focal incumbents participating in trial evaluations supported integration because it forced incumbents to justify their interpretations of an aspirant’s trial performance both to themselves and others, producing greater consensus in evaluation among incumbents. These two practices also supported adjustments by incumbents when the allocated role component was new to the group. For instance, an aspirant who demonstrated a more effective and reliable way to modify the surface texture of cooked vegetables immediately prompted several members of the group to change the sets of tasks they routinely performed to prepare ingredients for each day’s service because some of those tasks had been rendered obsolete by the aspirant’s new technique (observed at a restaurant in Europe).

Aspirants with industry experience were often said to bring in role components representing incremental process or conceptual innovations that stimulated adjustment within the group. But respondents distinguished these incremental innovations from the much-rarer disruptive innovations “from way out in left field . . . [that] profoundly, fundamentally changed how we worked” (I-49). Role components representing non-incremental innovations, respondents said, were more likely to come from aspirants who had work experience outside the culinary field (i.e., who were less experienced in terms of culinary skill but not necessarily always younger).

#### Group-level integration: Adaptation and change

For disruptive role components, respondents said that integration and adjustment occurred not just among aspirants and incumbents but for the group as a whole. This unintended but widely recognized side effect of negotiated joining was generated by aspirants when they managed either implicitly or explicitly to persuade incumbents that the group’s existing system of goals and roles was suboptimal for the external environment and that the integration of the aspirant’s disruptive role component (with attendant adjustments to incumbents’ roles and group goals) would be a change for the better. One incumbent echoed a majority of respondents when he told me that “[aspirants] sometimes come up with things that we would never have thought of ourselves that somehow are just right for what we like . . . sometimes, spending time with these new people who might join our team, it helps us to understand that they can bring us where we don’t even know we want to go yet” (I-52).

Incumbents framed these disruptive role components as new resources that could be combined with existing group resources in ways that had been previously inconceivable. It was a rare aspirant who brought in this type of role component that let the group “solve a problem that we didn’t know was good to solve before” (I-52), but nearly every senior incumbent could recall at least one instance of such a major change. For instance, an incumbent at a U.S. R&D lab reported that an aspirant’s set of newly assigned role components triggered “a new way of thinking that changed the way we thought about what we do as a group. Suddenly, we had [someone on the team who was a Ph.D.-trained physicist and chemist] and we could imagine working on things that needed that specialized knowledge” (I-2).

Because novel role components introduced by aspirants could fundamentally change group goals, respondents said that integration in these situations could be radical and affect many or even all incumbents in the group: “[Having a new person on the team like this] forces you to re-think your job. . . . [A current aspirant], for instance, is doing some biz dev for us. If he comes on board, we’ll be all about partnering to develop new products or starting a restaurant, but we were researchers before that so now we’re wondering how we can still be relevant, what our new jobs should be” (I-15). This adaptation was possible only because of the incumbent-centric adjustment and integration process and the assumption that a new member’s entry would likely trigger reconfiguration of incumbents’ roles.

## A Process Theory of Negotiated Joining

Negotiated joining consisted of proposition, trial, evaluation, and integration—four separate subprocesses associated with specific practices that, in sequence, allowed aspirants and target groups to mutually propose and validate role components, allocate them to aspirants, and integrate them into the existing role structure of the target groups.

**Figure 1.**
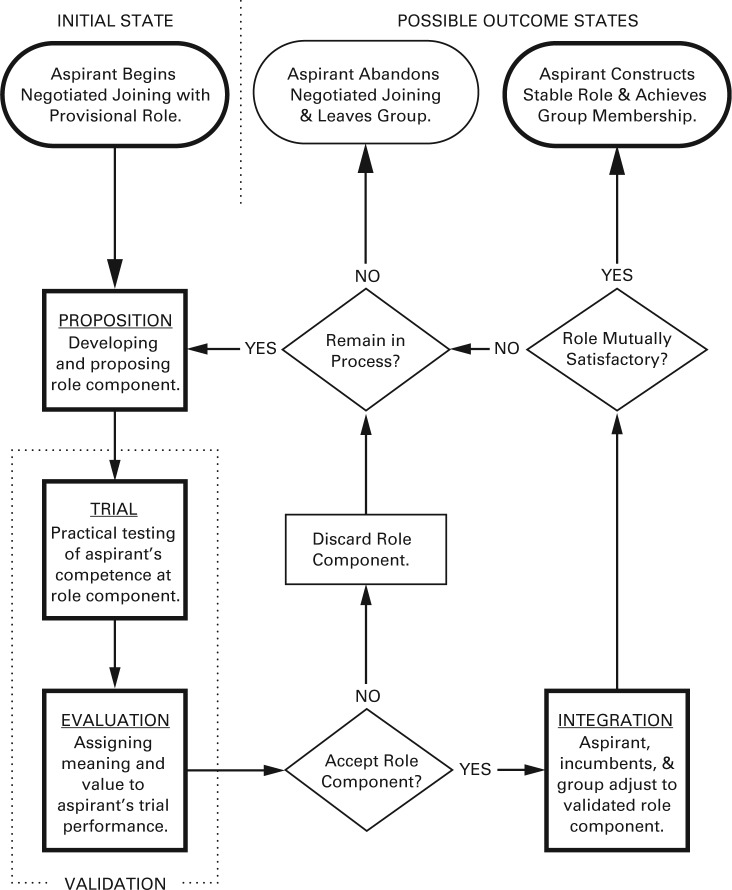
The iterative negotiated joining process, showing aspirant’s initial state, subprocesses, decision points, and aspirant’s possible outcome states.* * In this process diagram, lozenges indicate states, boxes indicate process events, and diamonds indicate decision points.

An aspirant’s progress through negotiated joining—both through the subprocesses of each iteration and through multiple iterations of the whole process—depended on decisions made at three points. The first occurred after evaluation, when the aspirant and the target group had to decide if the role component (and the aspirant’s performance of it) was mutually acceptable. The second occurred after integration, when again both had to decide if the aspirant’s role had become stable and mutually satisfactory with the addition of the new role component. If the aspirant’s role had not yet stabilized, both aspirant and target group had to make a third decision: whether the aspirant would remain in negotiated joining and initiate another iteration or abandon the process. Figure 1 illustrates the entire iterative negotiated joining process and shows the relationship between the subprocesses and the decision points in negotiated joining.

Selectively retaining only validated role components resulted in roles that were stable because they comprised role components that both the aspirant and the target group wanted the aspirant to perform: good role fit due not only to accurate selection by the target group (simple role fit) but also to a mutual process of role negotiation and role construction (constructed role fit). The undefined part of the aspirant’s role gained definition and stabilized as it “filled up” with validated role components. Yet even these roles were considered stable only in relation to aspirants’ initially provisional roles: incumbents said that it was normal for them to adjust their roles to integrate aspirants’ newly validated roles, and I observed this continual adjustment among incumbents at every field site.

### Enabling Conditions

Negotiated joining was universally said to be costly in time and effort for both aspirants and target groups. It was also potentially fraught: aspirants and incumbents had to work together for extended periods to construct aspirants’ roles and adjust incumbents’ roles, which should have resulted in turf battles between incumbents and aspirants. But respondents’ accounts and my observations suggested that negotiated joining was used at all sites for all aspirants despite its high cost and was regarded as unexceptional both at my field sites and in the industry as a whole. Moreover, extended role negotiation provoked few serious interpersonal or role-related conflicts. Though respondents all regarded negotiated joining as unsurprising, it seems reasonable to wonder what motivated aspirants and target groups to go through such an uncertain, time- and effort-intensive job search process, and how groups using negotiated joining managed to avoid collapsing under the burden of role-related conflict. Three conditions seemed to enable negotiated joining in this setting: (1) explicitly provisional initial roles, (2) framing outcomes as personal choices, and (3) prevailing norms and expectations.

#### Explicitly and mutually acknowledging the provisionality of initial roles

Role conflict can arise when an aspirant’s expectations about his or her role are violated or when an incumbent’s expectations about an aspirant’s role are violated. The explicit provisionality of the aspirant’s role minimized these expectations and seemed to remove this major potential source of conflict. A senior incumbent said that “people don’t get in a pissing contest about [the roles they perform] when they don’t really know what they’re supposed to be doing yet” (I-50). Provisionality also motivated the aspirant—respondents said that the possibility of a stable eventual role combined with the explicitly provisional nature of the initial role prompted aspirants to invest time and effort in negotiated joining processes.

#### Framing negotiated joining outcomes as personal choice

Respondents said that those who chose high-end culinary work generally had greater ambition and more advanced skills, giving them multiple employment options: “any one of the stagiaires we have working for free tonight could go off and run a kitchen at some two-bit club and make way more [money]. But they’re doing this because they want something more, right?” (I-38). Respondents consistently said that their top priority was “doing work worth doing in a restaurant worth working in” (A-27) and that “once you get used to [working with people of this quality] you can’t go back” (I-39). Consequently, they only described themselves as having significant attachment to roles that they had validated as being mutually desirable and were frequently “willing to walk away if it’s not the right place” (I-43). Respondents treated the considerable time and effort they spent on negotiated joining at various target groups as a worthwhile investment in eventually creating the right role for themselves there or elsewhere. This framing converted what might have been a point of interpersonal conflict—invalidation of a previously desired role or role component— into a less-conflictual personal choice.

#### Professional norms and expectations

Probably the main enabling condition in this setting is that high-end, cutting-edge culinary groups and workers are part of a community of practice in which the norm is for aspirants’ roles to be provisional and in which recruitment is almost universally managed through negotiation-dominant joining processes. My respondents thus used negotiated joining by convention, not by choice, generating the two enabling conditions explained above. Incumbents and aspirants both said that investing time and effort in negotiated joining was not seen as a deliberate decision but simply “how things are done in [this industry]” (I-22).

## Discussion

Previous studies take selection into predefined roles as the default model for systematically acquiring new members, consequently regarding incompletely predefined roles and other apparent departures from selection as idiosyncratic (Miner, 1987) or even irrational (Khurana, 2004). The groups I observed faced external uncertainty; their new members’ roles were necessarily open-ended, and these groups therefore could not and did not depend solely on selection processes to acquire new members. But the unsystematic, irrational, individualistic opportunistic hiring by managers predicted by previous theory did not ensue. On the contrary, these groups managed the acquisition of new members systematically using an iterative process new to the literature: negotiated joining.

The characteristics of negotiated joining described earlier reveal that it is distinct from selection as currently theorized. Table 4 summarizes these differences. Nonetheless, negotiated joining was systematic, consistently patterned, and allowed groups to achieve constructed role fit for new members with open-ended roles. While the pattern of practices associated with negotiated joining in elite culinary groups reported here is setting-specific and unlikely to generalize, the underlying negotiated joining process has broader applicability for managing membership in elite groups.

**Table 4.** Negotiated Joining Contrasted with Selection, from the Target Group’s Perspective

	Negotiated joining	Selection
Initial role	Provisional and open-ended.	Stable and predefined.
Objective	Figure out if mutually desirable role is likely and, if so, construct it.	Hire best-fitting aspirant to fill predefined role.
Types of fit desired	Constructed role fit, as well as cultural fit and simple role fit.	Cultural fit and simple role fit.
Method	Role construction through iterative role negotiation.	Linear selection against predefined criteria.
Successful outcomes	Learning about fit preferences; constructing a mutually desirable role.	Hiring aspirant with a good fit with predefined role.

### Generalizability

Negotiated joining’s distinctive characteristics and enabling conditions suggest that it is more likely to be observed where target groups and aspirants recognize that roles must be emergent and open-ended. This is the case for groups that experience external uncertainty, which makes members’ roles difficult to predefine. Additionally, aspirants and target groups must be willing and able to allocate large amounts of time and effort to role negotiation. Large payoffs from group membership allow groups to have surplus resources to spend on negotiated joining and also incentivize aspirants to attempt to join these groups. Similarly, high-quality aspirants who are in short supply are more likely to be able to invest time and effort in negotiated joining, and groups are more likely to be incentivized to invest resources in constructing roles around them.

These three conditions—necessarily open-ended roles, large payouts from group membership, and high-quality aspirants in short supply—characterize elite groups that must be staffed from elite-aspirant pools and that face uncertain future demands. Groups in this category include prestigious academic faculties seeking high-quality scholars for tenure, professional service firms seeking new equity partners, and top management teams in new or rapidly changing industries seeking new senior management. The process theory developed here suggests that these elite internal groups are likely to acquire new members from their aspirant pools using negotiation-dominant processes marked by initially provisional roles and iterative role-negotiation through proposition, trial, evaluation, and integration.

By the same reasoning, negotiated joining is too costly and uncertain to be the primary mode of new-member acquisition in all contexts. Groups with predefinable roles operating in relatively stable environments, with minimal organizational slack, and with an abundance of qualified aspirants—for instance, groups performing basic customer service work (e.g., Fernandez and Sosa, 2005) or manufacturing assembly work (e.g., Hossfeld, 1993)—have neither the capacity nor the incentive to use negotiated joining. Selection-dominant processes are both more appropriate and more likely to be observed in such settings.

### Contributions

#### Membership dynamics in elite groups

Current research in this area focuses on group, individual, and role characteristics that predict membership (Rivera, 2012, 2014) and subsequent member performance (Groysberg, Lee, and Nanda, 2008; Groysberg and Lee, 2009) in elite groups. My findings show that this research agenda should be expanded to also examine the processes and dynamics that lead to membership in internal elite groups. The example of management consulting firms is illustrative. While these consulting firms are usually considered elite organizations in their own right (Rivera, 2014), equity partnership groups within these firms represent an even more exclusive type of group. Equity partners have relatively stable roles and domains of expertise; by contrast, pre-partnership employees have provisional roles (they are employed understanding that they either move “up or out”) and should be considered potential aspirants to partnership. Negotiated joining helps make sense of the pre-partnership trajectory, in which an aspirant must develop a “platform” for election to partnership: domain expertise comprising a unique configuration of role components and individual client relationships that takes advantage of the aspirant’s skills and inclinations and is relevant to the firm’s needs. These role components are validated through multiple client engagements and interactions with partners and other colleagues over the course of many years. Attrition rates are high, and examining these setting-specific negotiated joining practices may better explain who becomes a partner at these firms and why.

Additionally, my findings suggest that the more dominant negotiated joining is in these elite groups’ membership processes, the more likely that initial roles in these groups will be provisional and stable roles will be customized to fit a combination of group needs and individual inclinations. Negotiated-joining theory predicts a performance benefit for target groups that offer aspirants more opportunities to mutually construct their roles, because these groups will be more likely to acquire new members who perform well.

#### Scope conditions for beneficial role ambiguity

Negotiated joining also updates previous research that uniformly construes role ambiguity as being unpleasant for the focal individual, detrimental for group performance, and generally undesirable (Rizzo, House, and Lirtzman, 1970; Ilgen and Hollenbeck, 1991; Tubre and Collins, 2000; Dierdorff and Rubin, 2007). In the present study, initial roles were ambiguous because aspirants and target groups mutually and explicitly agreed on their provisionality. This provisionality allowed roles to be constructed that were desirable to both the aspirant and the target group and sometimes allowed aspirants to import novel, disruptive role components into their target groups. As described earlier, these disruptive role components changed group goals and role structures, and respondents reported that these internal changes allowed groups to adapt to changing demands from the external environment. Explicitly ambiguous roles, such as provisional roles, could generate good role fit through construction as well as promote group adaptation to external uncertainty. The present study suggests a scope condition for role ambiguity by showing that symmetrically explicit role ambiguity can be beneficial for both individuals and groups.

### A Contingency Theory of New-member Acquisition

Negotiated joining has implications beyond extending our understanding of the membership dynamics of elite groups and the potential benefits of role ambiguity. The dominance of selection in previous research has led to the generally unquestioned assumption that systematic new-member acquisition is a normatively and descriptively non-contingent process. The existence of negotiated joining as a viable alternative to selection calls this assumption into question and demonstrates the utility of a contingency theory of new-member acquisition. While previous theory has called for incorporating environmental assessment into the process of strategic job analysis as a precursor to applying selection methods (Singh, 2008), I argue that the method of new-member acquisition itself should be contingent on the nature of the environment.

Contingency theories focus on the fit between organizational characteristics and organizational environments and use degree of fit to explain organizational performance (Van de Ven, Ganco, and Hinings, 2013). In this case, fit is between how an organization acquires new members and the degree of predefinability of new members’ roles, while performance is the organization’s continued ability to survive and flourish by adapting to the demands of the environment.

In stable environments, actions map consistently to outcomes; organizations can therefore learn from experience and optimize their processes and structures (Sorenson, 2003) by developing consistent roles and routines (Levitt and March, 1988). In contrast, action–outcome mappings change unpredictably in unstable, uncertain environments; roles in organizations operating in such environments are necessarily emergent and open-ended. Under these latter conditions, a rigid structure of roles and routines is suboptimal (Burns and Stalker, 1961). Instead, organizations and their members must have adaptability—the dynamic capability to detect external change and change internally in response (Teece, Pisano, and Shuen, 1997; Arrow, McGrath, and Berdahl, 2000; Lewin, Massini, and Peeters, 2011)—to survive and flourish in uncertain environments (Argote and McGrath, 1993; Miron-Spektor, Gino, and Argote, 2011).

Negotiated joining both accommodates open-ended roles and can promote adaptability by stimulating ongoing adjustments to group structure and goals, which is an unintended side effect of integrating newly validated role components. Negotiated joining therefore enables organizing through emergent relationships within groups (Stewart and Carson, 1997) and serves as a previously unidentified mechanism for increasing absorptive capacity (Zahra and George, 2002; Lewin, Massini, and Peeters, 2011) by providing ongoing opportunities for target groups to adapt by absorbing novel skills and information that aspirants bring in from the external environment.

Whereas previous research has assumed and prescribed that selection is the sole and normatively preferable method for acquiring new members, this study introduced external uncertainty and the resulting difficulty in predefining new members’ roles as a condition that affects an organization’s choice of new-member-acquisition processes. Negotiated joining and selection are not mutually exclusive, and my findings show that selection and negotiated joining can be used in combination: no group I observed relied exclusively on negotiated joining, and every group relied primarily on negotiated joining supplemented with conventional selection processes such as résumé reviews and interviews. Consequently, in a contingency theory of new-member acquisition, the appropriate balance between selection and negotiated joining depends on the level of uncertainty in the external environment: more uncertain environments require less-predefined, more open-ended roles and call for more negotiation-dominant membership management processes.

### Limitations and Future Research

This paper set out to document the understudied phenomenon of negotiated joining in the setting of elite cutting-edge culinary work. To do so, I examined established groups in this setting and focused on finding similarities across cases. It is therefore beyond this paper’s scope to explain variations in process outcomes across groups or individuals. To extend our understanding of how negotiated joining works, future research should explore the phenomenon in other settings and pose questions that this study did not address.

Elite cutting-edge culinary groups were an ideal research setting in which the general process mechanism of negotiated joining was clearly observable. But because negotiated joining happens in the course of routine work interactions, the specific practices that support negotiated joining are likely to vary considerably across settings. For example, a venture capital firm seeking a new general partner is likely to use very different negotiated joining practices than an academic department seeking a new tenured faculty member. Future research could document negotiated joining practices in different work and professional settings to explore areas of similarity and difference. The contingency model of new-member acquisition developed above indicates that these efforts should focus especially on settings marked by high uncertainty.

The type and amount of data I collected—interviews and short-duration observations—prevented me from examining how and to what extent negotiated joining affected individual and group outcomes beyond what respondents reported. Respondents said that negotiated joining produced roles that were mutually desirable, implying greater job satisfaction and longer tenure. They also said that negotiated joining could stimulate group adaptability, implying greater group longevity. Future research should explicitly test these propositions. One possible study could compare groups using negotiation-dominant member acquisition processes with groups using selection-dominant processes to see if there are systematic differences in group longevity and in members’ tenure and satisfaction. Another study could investigate individual and group outcomes associated with negotiated joining using longer-term observations of groups and aspirants. Also, recent research on new ventures in an uncertain environment shows that new firms with more formal structures (and thus more clearly predefined roles) outperform those with less formal structures (Sine, Mitsuhashi, and Kirsch, 2006)—a third study could examine if and how the effects of negotiated joining vary across organizations at different stages of development.

Finally, I focused on negotiated joining as a membership process used by target groups, but my respondents were worthy of study as well: they were uniformly willing to accept provisional roles and the high likelihood of not constructing stable roles for themselves. Respondents indicated that unsuitable individuals would self-select out, which could suggest systematic differences between individuals who are effective participants in negotiated joining and those who are not. Personality is probably part of the explanation: individuals oriented toward uncertainty and open to new experiences (Hodson and Sorrentino, 1999) will be more likely to embrace the uncertainty in negotiated joining, and those who are relatively high in conscientiousness and emotional stability (Barrick and Mount, 1991; Stewart and Carson, 1997; Le et al., 2011) will be more likely to handle the interactions involved in negotiated joining gracefully. The aspirants in this study were drawn from elite pools of individuals with high ability and flexibility, consistent with previous theorizing that organizing around emergent relationships would be associated with individuals who had greater general mental ability (Stewart and Carson, 1997). A research program could thus focus on the individual level to identify personality and other characteristics that make individuals more or less likely to be predisposed to participate in negotiated joining rather than looking for predefined roles they can fill.

## Footnotes

1 I = an incumbent in a target group and A = an aspirant who hopes to join the target group; the number following denotes an individual respondent in this study. I-46 is the 46th of 55 incumbents.
